# In-situ development of a sandwich microstructure with enhanced ductility by laser reheating of a laser melted titanium alloy

**DOI:** 10.1038/s41598-020-72627-x

**Published:** 2020-09-28

**Authors:** Xu Chen, Chunlei Qiu

**Affiliations:** School of Materials Science and Engineering, Beihang University, Beijing, 100191 China

**Keywords:** Materials science, Structural materials, Engineering, Aerospace engineering

## Abstract

Metallic additive manufacturing, particularly selective laser melting (SLM), usually involves rapid heating and cooling and steep thermal gradients within melt pools, making it extremely difficult to achieve effective control over microstructure. In this study, we propose a new in-situ approach which involves laser reheating/re-melting of SLM-processed layers to engineer metallic materials. The approach involves alternate laser melting of a powder layer at a high laser power and laser reheating of the newly formed solidified layer at a low or medium laser power. This strategy was applied to Ti-6Al-4V with a range of laser powers being used to reheat/re-melt solidified layers. It was found that the SLM-processed sample without undergoing laser reheating consist of a pure martensitic needle structure whereas those that were subjected to laser reheating/re-melting all consist of horizontal (α + β) bands embedded in martensitic α′ matrix, leading to development of a sandwich microstructure in these samples. Within the (α + β) bands, β exist as nano-sized precipitates or laths and have a Burgers orientation relationship with α matrix, i.e., {0001}⍺//{110}β and ⟨11$\stackrel{-}{2}$0⟩⍺//⟨111⟩β. The width of (α + β) banded structure increased first with increased laser power to a highest value and then decreased with further increased laser power. With the presence of these banded structures, both high strengths and enhanced ductility have been achieved in the SLM-processed samples. The current findings pave the way for the novel laser reheating approach for in-situ microstructural engineering and control during metallic additive manufacturing.

## Introduction

Metal additive manufacturing (AM) is a melt-pool-based additive process which involves line scanning and layer accumulation, thus demonstrating a powerful near-net-shape manufacturing capacity in fabricating components or structures with intricate geometries or customised designs. Among various metal AM processes, direct laser deposition (DLD), selective electron beam melting (EBM) and selective laser melting (SLM) are the three most commonly used processes. The DLD process usually has a wide range of laser powers (up to 10 KW) and powder/wire feeding rates, which allows it to produce melt pools with various sizes (from several hundred micrometers up to several millimeters) and thermal conditions^1–4^. This endows the DLD process with a good microstructural controllability. Both non-equilibrium and equilibrium microstructures can be produced in DLD-processed Ti-6Al-4V^1–3^. Significant columnar-to-equiaxed grain transition (CET) can be achieved in a number of (α + β) titanium alloys^4–6^. Moreover, DLD can be easily fitted with other affiliated facilities such as a roller or an ultrasound facility to further enhance its capability in microstructural control^7–9^. EBM operates at a medium laser power (~ 1 KW) and has relatively lower process flexibility as compared with DLD. CET could be hardly observed in EBM-processed titanium alloys. Nonetheless, with its powerful preheating capacity through electron beam raster scanning (> 700 °C), an equilibrium α + β microstructure can be easily achieved in EBM-processed Ti-6Al-4V^10–12^. As compared with DLD and EBM, SLM has neither a large power range nor a powerful preheating capability. As a result, metallic SLM usually ends up with highly small melt pools (several hundred microns in diameter), highly rapid cooling and steep thermal gradients in melt pools, which easily lead to development of columnar grains and texture and non-equilibrium microstructure^13–16^. The typical microstructure in SLM-processed Ti-6Al-4V is well developed columnar prior β grains and non-equilibrium martensitic needle structure^17–20^. The martensitic needle structure usually gave rise to high strengths but significantly reduced ductility^18–20^. As a result, a post-SLM heat treatment or HIP (hot isostatic press) was often used as a routine to produce equilibrium α + β microstructure^18,21–24^, which was beneficial to ductility but at the remarkable cost of strengths due to the development of much coarsened α + β lamellar structure. The ideal situation is to produce a refined α + β microstructure so that both high strengths and excellent ductility can be obtained. Recently, Xu et al.^25,26^ successfully in-situ developed ultrafine lamellar α + β microstructure in SLM-processed Ti-6Al-4V alloy by carefully selecting a combination of a number of processing parameters including laser power, scanning speed, powder layer thickness, inter-layer time, hatch spacing, part diameter, area ratios between support structure and part. The fine lamellar α + β microstructure not only yielded a tensile elongation of 11% but also maintained a high yield strength above 1100 MPa. However, it is noted that the necessity for stringent control on so many processing parameters (particularly the part diameter and the area ratios between support structure and parts) to achieve the α + β microstructure constitutes a great limitation for this approach to be adopted widely in industrial-scale manufacturing.

**Figure 1:**
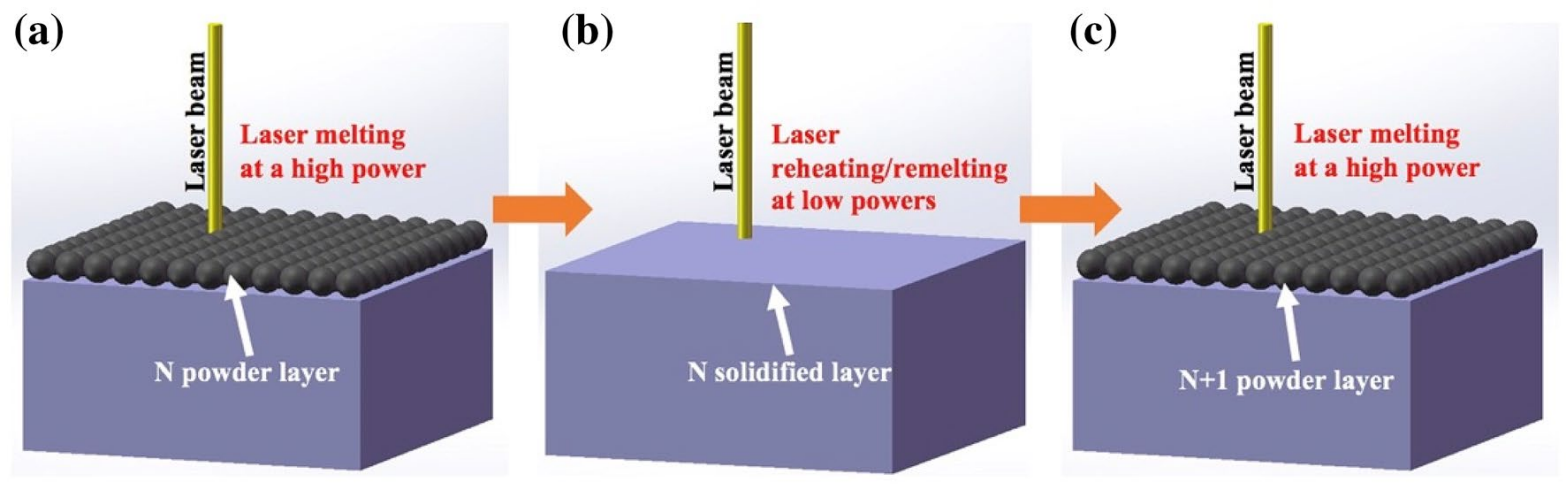
Schematic illustration of (**a**) and (**c**) powder laser melting and (**b**) laser reheating/re-melting process.

So far, there is a lack of effective means to engineer and tailor microstructure during SLM. It is thus necessary to explore a new, simple and effective route to promote microstructural control and engineering during metallic SLM. In this study, we propose a novel and simple in-situ approach to engineer microstructure of metallic materials during SLM. The new approach involves laser reheating on newly built layers to introduce an in-situ rapid heat treatment on the SLM-processed layers. Specifically, each powder layer will be first SLM processed at a high laser power to form a fully consolidated layer which will then be immediately reheated/re-melted by a laser beam with a low or medium laser power (illustrated in Fig. 1); this process will then be repeated until a complete sample or part is built. To be noted, a double or multiple laser scanning strategy has been adopted in several previous studies to re-melt previous laser processed layers with high laser powers (normally several hundred W) to improve either surface quality, or inter-layer bonding or change texture in materials^27–29^. In the current study, our approach is not aimed for re-melting but more for re-heating to promote solid state phase transformation. As a result, several low laser powers (< 100 W) will be used for laser reheating. Some relatively higher laser powers (100–200 W) which may cause partial re-melting of previous layers will also be tried as a comparison. This approach will be applied to Ti-6Al-4V to validate its feasibility and effectiveness in tailoring microstructure. The influence of laser reheating/re-melting on microstructural development and tensile properties will be thoroughly investigated.

## Results and discussion

### Porosity and microstructural development

**Figure 2:**
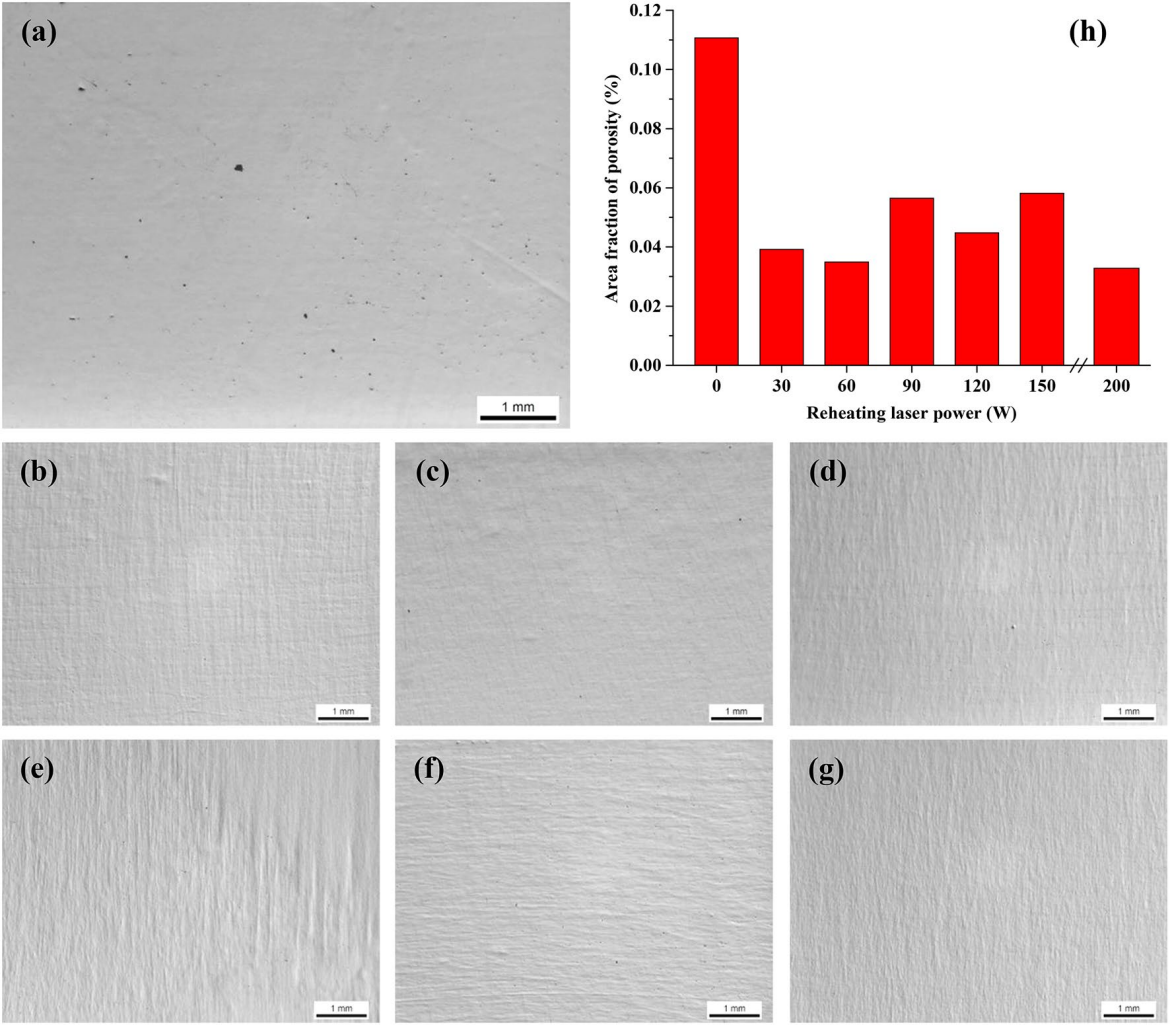
OM micrographs showing the porosity distribution in the samples that were processed at 400 W (**a**) without reheating and with reheating at (**b**) 30 W; (**c**) 60 W; (**d**) 90 W; (**e**) 120 W; (**f**) 150 W; (**g**) 200 W. (**h**) Measurement statistics of area fractions of porosity in samples reheated at different laser powers. The measurement scatter of porosity in these samples is within ± 0.01%.

**Figure 3:**
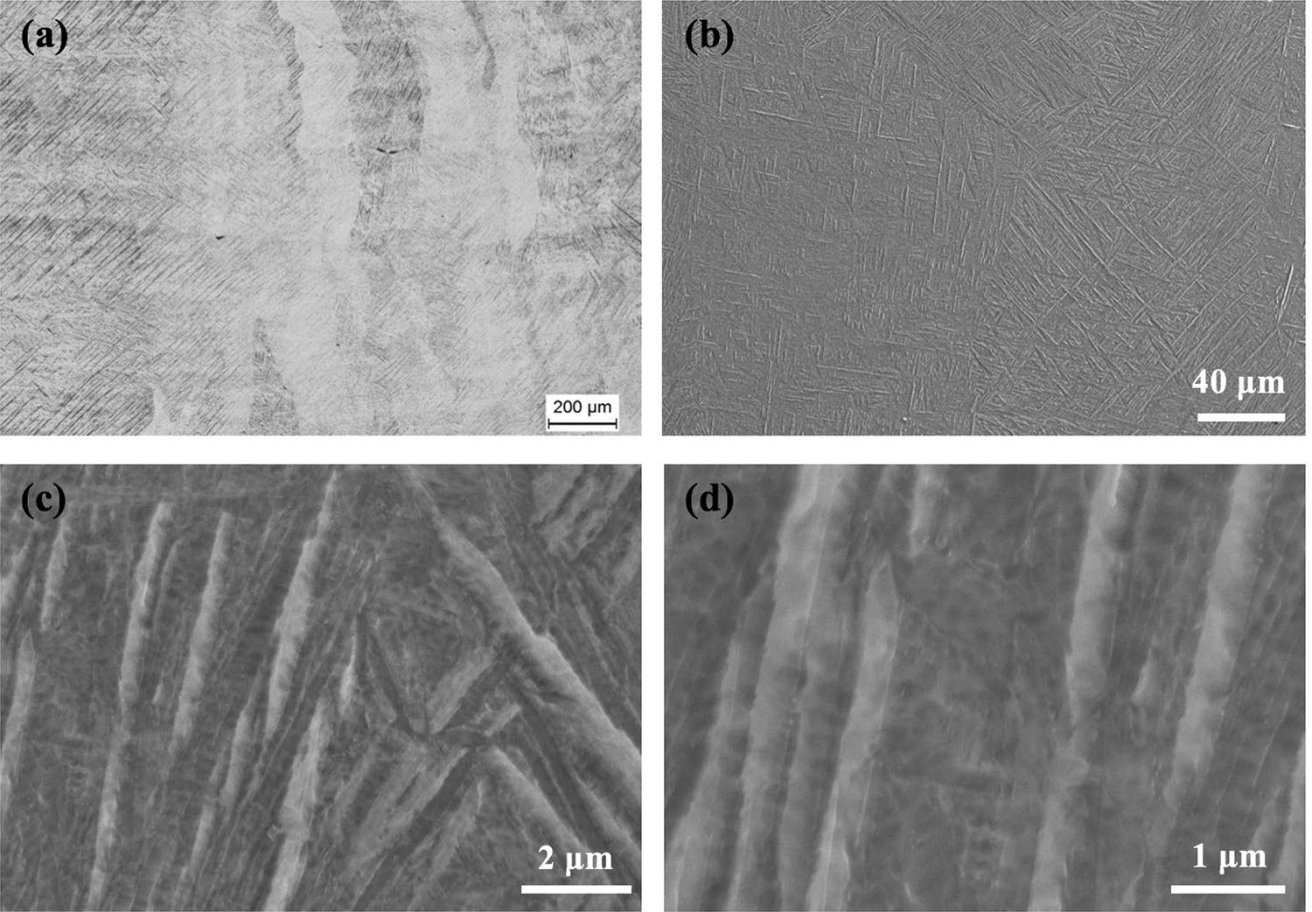
(**a**) OM and (**b**–**d**) SEM micrographs showing the microstructure of the sample that was made at 400 W.

Figure 2 shows the distribution and levels of porosity in the samples that were SLMed at 400 W with and without laser reheating. It is obvious that the sample made without reheating shows the highest porosity level (~ 0.11%) which is still at a very low level. Laser reheating or re-melting seems to be benign to consolidation as the porosity levels all reduce to more or less extents. Only a few small pores can be observed in the laser reheated samples; see Fig. 2b–g. The current results are consistent with previous studies^27,28^ where double or multiple laser scanning was reported to reduce porosity by improving the surface structure of previous layers as well as the interlayer bonding. Figure 3 shows the microstructure of as-SLMed Ti-6Al-4V sample that was simply processed at 400 W. It is clear that the sample is dominated by columnar prior β grains and martensitic needles. Many of the martensitic needles show bright colour under SEM, which could be attributed to the super-saturation of β forming elements such as V in the martensite. There is no obvious sign showing the decomposition of martensite in the as-fabricated state, which is consistent with the observation in many previous studies^17–19^.

**Figure 4:**
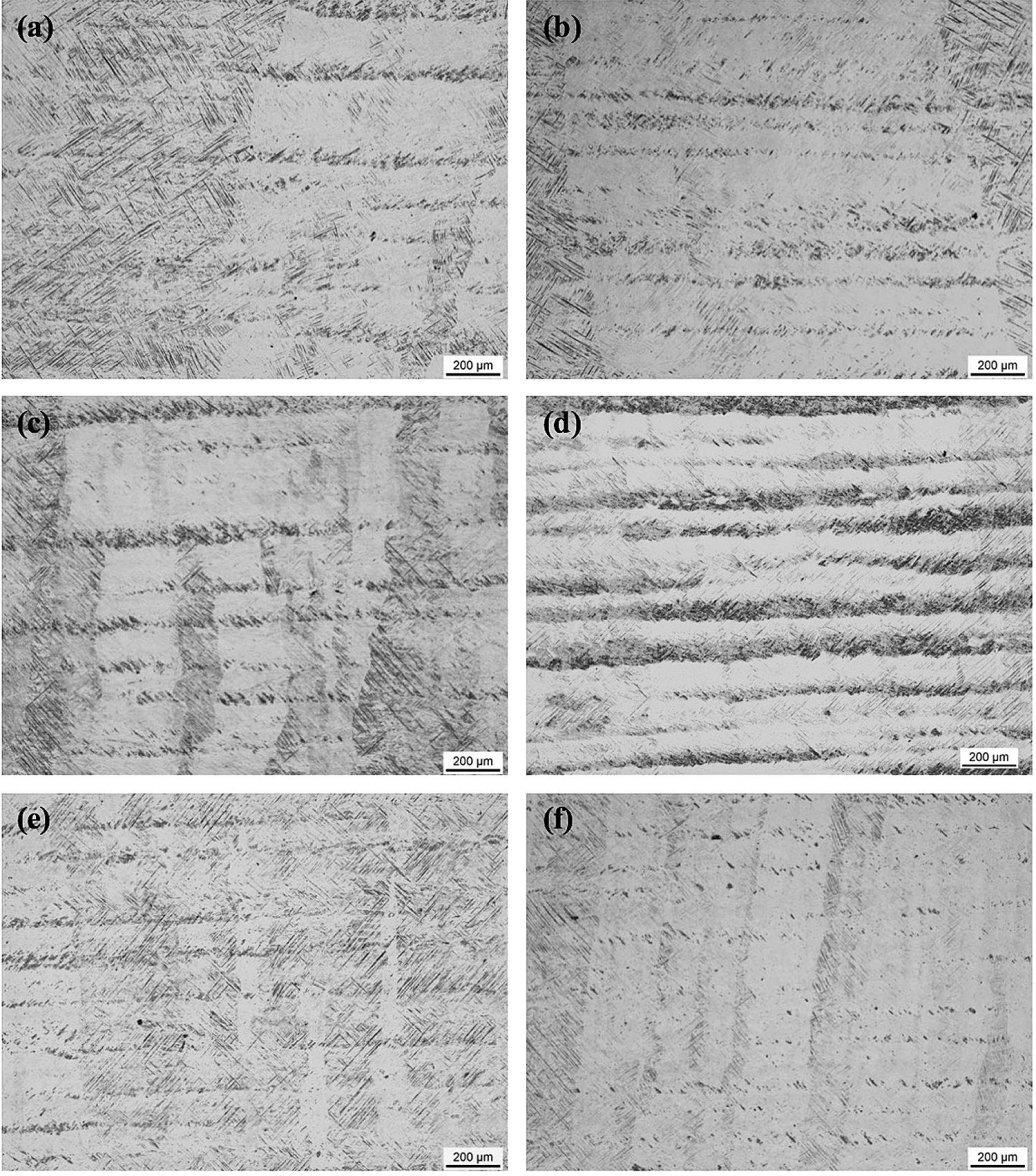
OM micrographs showing the microstructure of the samples that were processed at 400 W and reheated at (**a**) 30 W; (**b**) 60 W; (**c**) 90 W; (**d**) 120 W; (**e**) 150 W; (**f**) 200 W.

**Figure 5:**
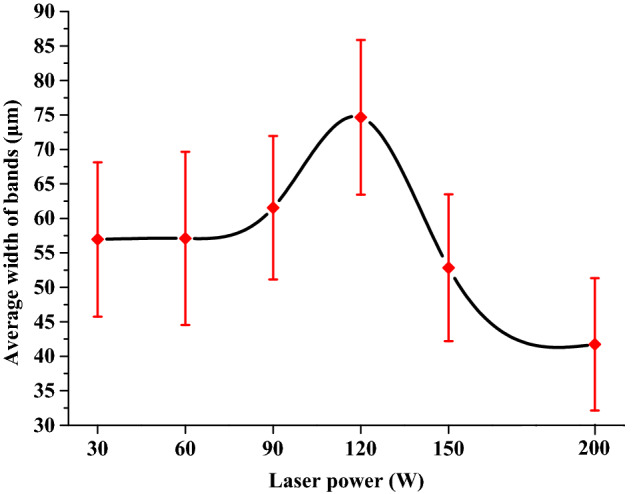
Dependence of average width of horizontal bands on laser power.

**Figure 6:**
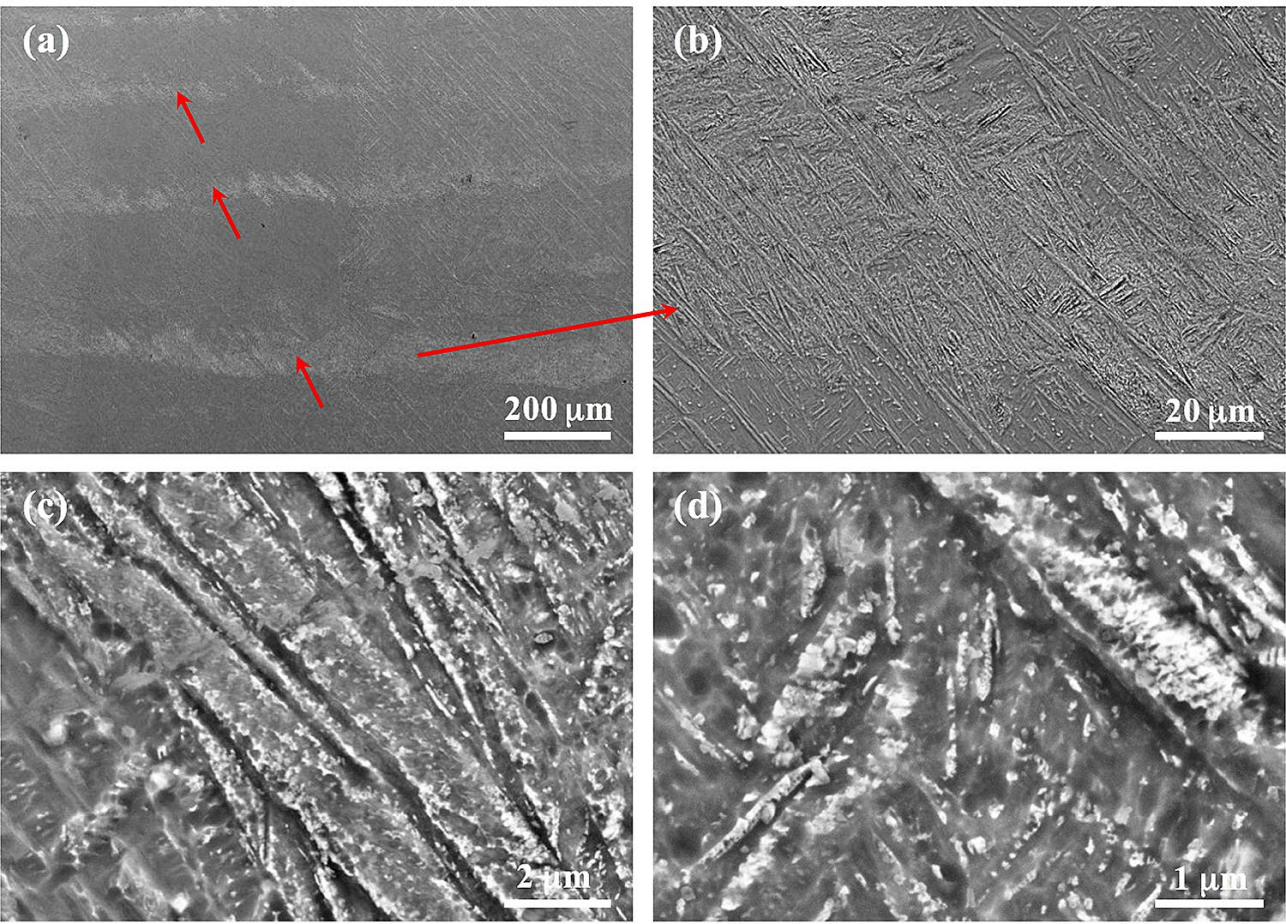
(**a**–**d**) SEM micrographs showing the microstructure of the sample that was processed at 400 W and reheated at 120 W on a layered basis. (**a**) alternate (α + β) and α′ banded structures and (**b**)–(**d**) high magnification SEM micrographs showing the details within a (α + β) band. The arrows in (**a**) show the banded regions which are enriched in fine precipitates.

**Figure 7:**
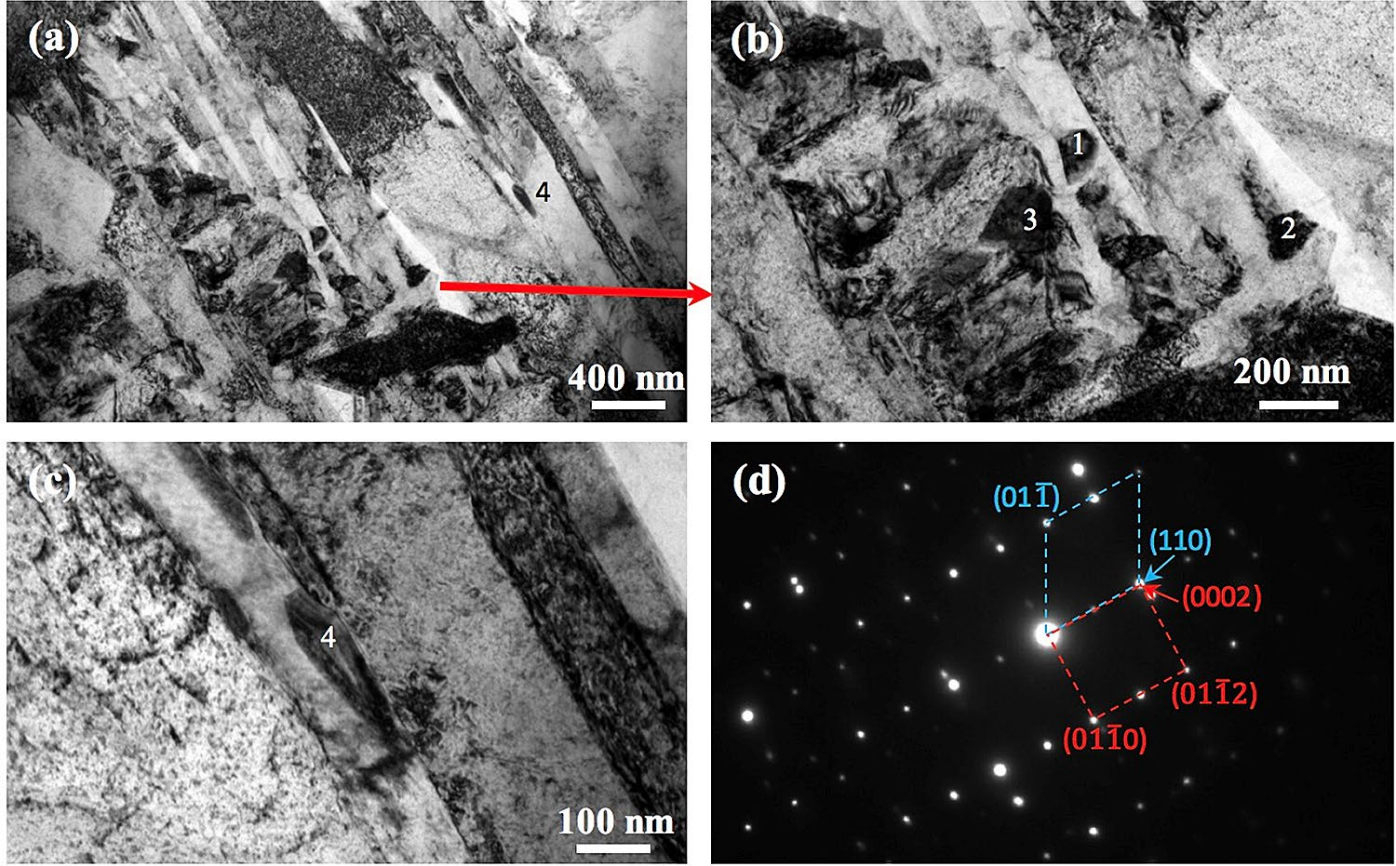
(**a**–**c**) Bright-field TEM micrographs showing the presence of small precipitates in the matrix; (**d**) TEM diffraction pattern for Precipitate 4 in (**c**) and part of the surrounding matrix. The α and β phases have a Burgers orientation relationship of {0001}⍺//{110}β and  ⟨11$\stackrel{-}{2}$0⟩⍺//⟨111⟩β.

**Figure 8:**
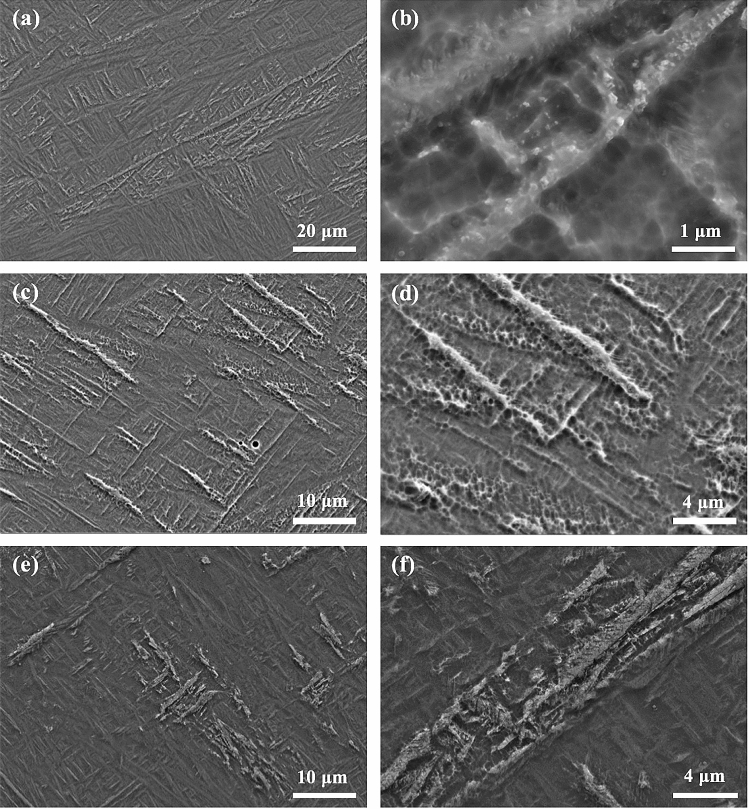
SEM micrographs showing the microstructure of the samples that were processed at 400 W and reheated at (**a**,**b**) 30 W; (**c**,**d**) 90 W; (**e**,**f**) 200 W.

**Figure 9:**
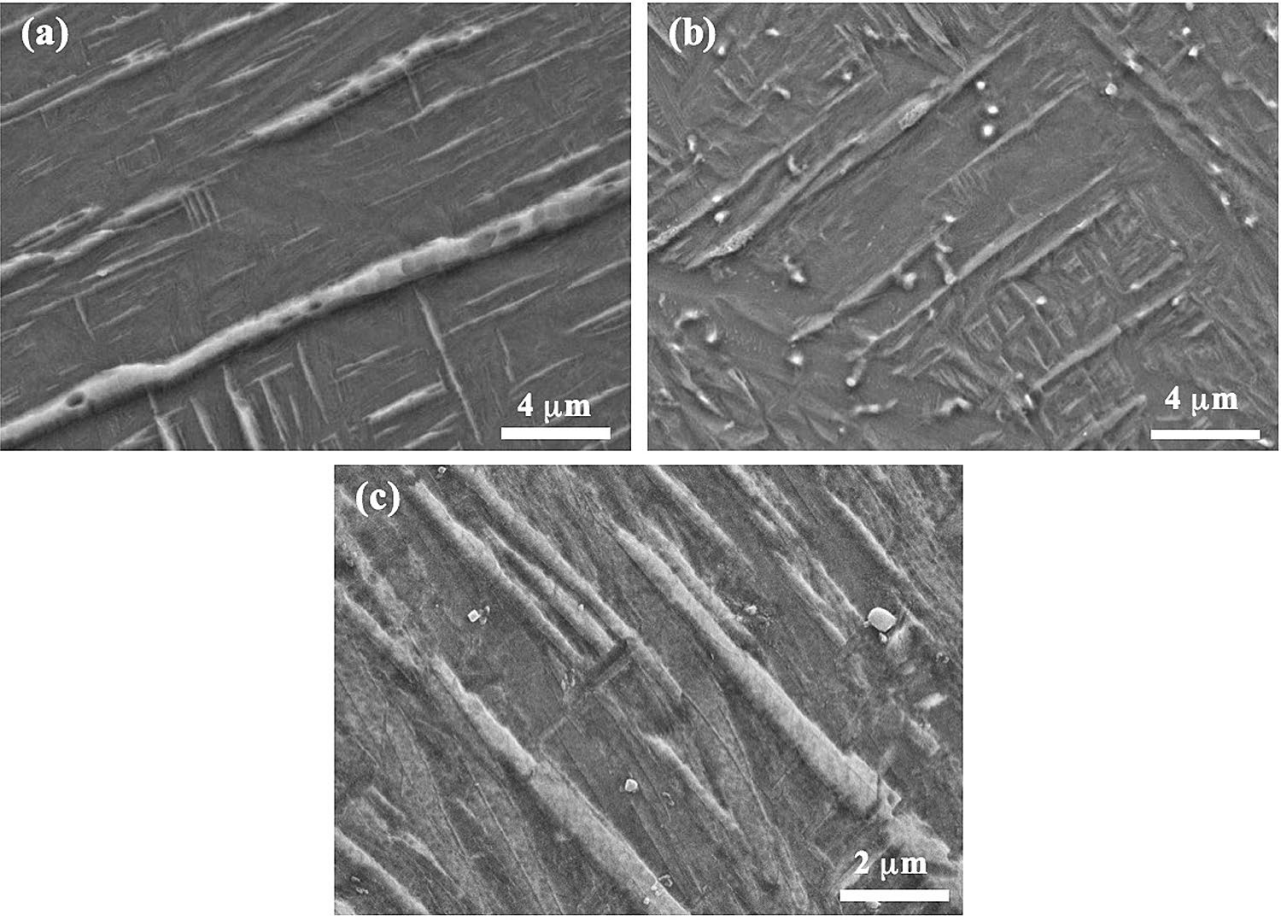
SEM micrographs showing the microstructure in the matrix areas of the samples that were processed at 400 W and reheated at (**a**) 30 W; (**b**) 120 W; (**c**) 200 W.

Figure 4 shows the microstructure of Ti-6Al-4V samples that were first SLM-processed at 400 W and then laser reheated at different powers on a layer basis. It is obvious that all the samples are still dominated by columnar prior β grains, indicating that the layered reheating/remelting treatment does not affect the grain morphology. However, all these samples contain a number of horizontal black bands (observed under OM) which are embedded in the matrix, leading to development of a sandwich microstructure. Within the reheating laser power range of 30–200 W, the width of the bands first increases steadily with increased laser power to a maximum value at 120 W from which the width of the bands decreases with further increased laser power; see Figs. 4, 5. The bands in most of the samples are generally around 50 μm in width, which is comparable to a powder layer thickness (50 μm). The maximum average band width which was observed in the sample reheated at 120 W can reach 75 μm. Figure 6 shows the detailed microstructure in some bands of the sample that was reheated at 120 W. It is clear that these bands turn bright under SEM and are characterised by the presence of massive ultrafine white precipitates either in particulate or lamellar form. This is further confirmed by TEM imaging results as shown in Fig. 7a,b where a number of precipitates are present within or between α laths. TEM–EDX analysis on some of these precipitates reveals that they are lean in Al but rich in V (Table 1), suggesting that these precipitates should be β phase. TEM diffraction analysis on one of such precipitates (Fig. 7c) and part of its surrounding matrix reveals that these precipitates are indeed β phase (see Fig. 7d). Moreover, it can be seen that the β precipitates and its surrounding α matrix have a Burgers orientation relationship of {0001}⍺//{110}β and  ⟨11$\stackrel{-}{2}$0⟩⍺//⟨111⟩β. It is also noted that almost all the martensitic needles in the bands of the sample reheated at 120 W have transformed into α laths and β precipitates. White bands and β precipitates are also observed in the other reheated samples but the precipitates are less dense than those in the sample that was reheated at 120 W; see Fig. 8. Moreover, there are still a number of fine martensitic needles present in the bands suggesting that in these bands the martensites have not completely turned into α + β. As a result, the bands are literally a mixture of ultrafine α + β + α′ (see the left column in Fig. 8), leading to development of a hybrid microstructure in these bands. In the matrix area that is away from the bands, the microstructure is purely dominated by martensitic needles in all the samples except for the one that was reheated at 120 W where some near-spherical white precipitates are distributed in the matrix; see Fig. 9. It is thus concluded that laser reheating at 120 W is most favourable for decomposition of martensitic needles and development of (α + β) bands in the SLMed Ti-6Al-4V samples.

**Table 1:** Chemical composition (at.%) of some precipitates shown in Fig. 5e,f obtained by TEM–EDX analysis.

Element	Particle 1	Particle 2	Particle 3	Particle 4
Al	2.4	2.9	2.9	2.8
V	3.8	3.8	4.1	4.9
Ti	Bal	Bal	Bal	Bal

The formation of β could be due to either the transformation of the entire local microstructure from α′ to β when the local reheating temperature was above the β transus temperature, or the sub-transus decomposition of the α′ to α + β microstructure. The former mechanism may not hold true for the current pulsed laser reheating condition which involves rapid heating and cooling (≥ 10^4^ °C/s). It has been shown that heating at sufficiently high heating rates could, in fact, suppress the α∕α′ → β transformation^30,31^. Kelly et al. measured the increases in the β transus as a function of temperature and observed that the β transus shifted by 30 °C for every 10 °C/s increase in the heating rate. Conservatively estimated, additive manufacturing involves heating rates of approximately 1000 °C/s, which could possibly cause the β transus to increase by as much as 300 °C from 995 °C which is the β transus when heating rates are low^31^. Even if all α′ transforms into β grains, during subsequent rapid cooling, they would completely transform into α′ again. This is because when the cooling rate from single β zone is higher than ≥ 410 °C/s^32^, martensite would form from β grains. The cooling rates after laser processing obviously are much higher than the critical martensitic formation temperature (≥ 10^4^ °C/s)^13–16^, which means β grains would transform completely into α′. Thus, the first mechanism should not be the reason for the development of (α + β) or (α + β + α′) banding structures in the current samples. For the second mechanism, it is noted that the α′ decomposition starting temperature for SLM-processed Ti-6Al-4V is around 700 ºC^18,23^. Obviously, to form β from the martensitic matrix, the laser reheating temperature should be at least beyond 700 °C but lower than the β transus which should be above 995 °C as described above. This is possible during laser reheating given that it can create a heat affected zone with a thermal gradient. There will always be some regions which fall in the sub-transus α′ decomposition temperature zone. However, it is noted during cooling at or above 410 °C/s, the starting martensitic formation temperature is 650 °C^33,34^. This means theoretically, β that forms above 650 °C due to reheating-induced α′ decomposition should completely transform into martensite during subsequent cooling. If this were the case, no β should have been observed in the current samples. The presence of β in the current laser reheated samples clearly indicates that the β formed during rapid reheating has not completely transformed into martensite during the subsequent rapid cooling. It is noted that a number of previous studies^35,36^ reported when the sizes of the parent phase were on a nanometer scale, the temperature of martensitic transformation upon cooling may dramatically decrease. This is probably the reason why β remains in the current laser reheated samples. Rapid laser reheating could have created a heat affected zone with a temperature range from 700 °C to β transus that was favourable for α′ decomposition but because of rapid heating, the duration for α′ decomposition and coarsening of β was highly limited. As a result, β remained as ultrafine nano-sized precipitates or laths, which may have significantly reduced the martensitic transformation temperature due to the size effect reported in the previous studies^35,36^ and thus effectively suppressed β → α′ transformation during rapid cooling. This allowed the ultrafine nanosized β precipitates to be retained to low temperatures, as has been observed in Figs. 6, 7, 8. For those re-melted regions, large β grains would inevitably form during solidification; for the regions that were heated above β transus, large β grains would also form. The β grains are so large (at the scale of hundred of micrometres) that they would not have the similar size effect as nanosized β. As a result, the martensitic formation temperature would not be compromised and the β grains could only transform into martensitic needles during rapid cooling. With a medium laser reheating power such as 120 W, a maximum heat affected zone that was suitable for α′ decomposition may have been created which allowed the maximum solid α′ decomposition zone to be formed. With increased laser power, more of the input energy may have been consumed in re-melting materials instead of creating a desirable heat affected zone for α′ decomposition. With a too low laser power, the heat affected zone may be too small to promote widespread α′ decomposition. Further investigation is needed to understand the phase transformation mechanisms during rapid laser heating and cooling.

### Tensile properties

**Figure 10:**
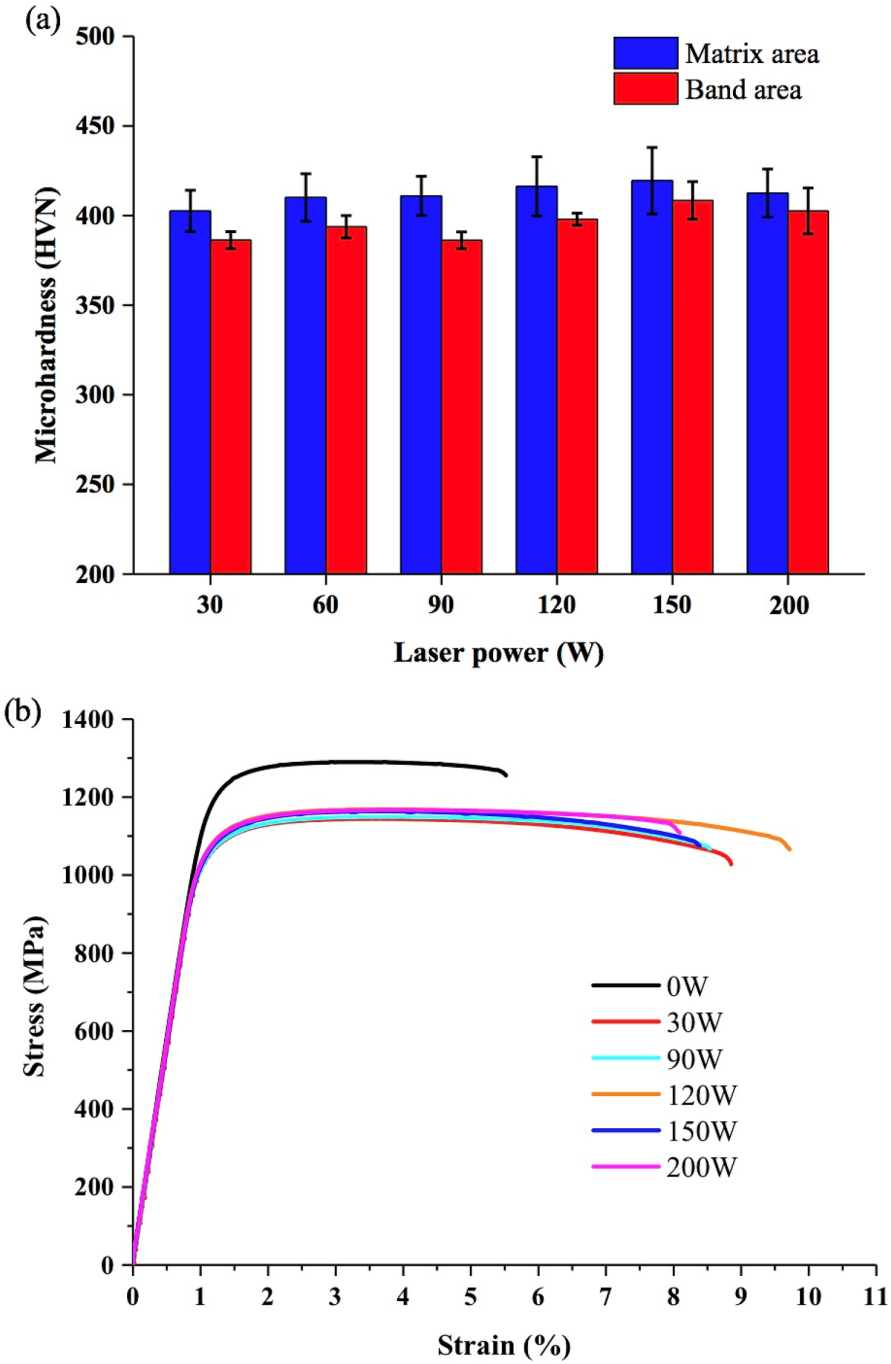
(**a**) Micro-hardness of the matrix and (α + β)-rich banding areas in the SLMed + reheated/re-melted samples; (**b**) Tensile stress–strain curves of the samples that were processed at 400 W and reheated at different laser powers.

**Figure 11:**
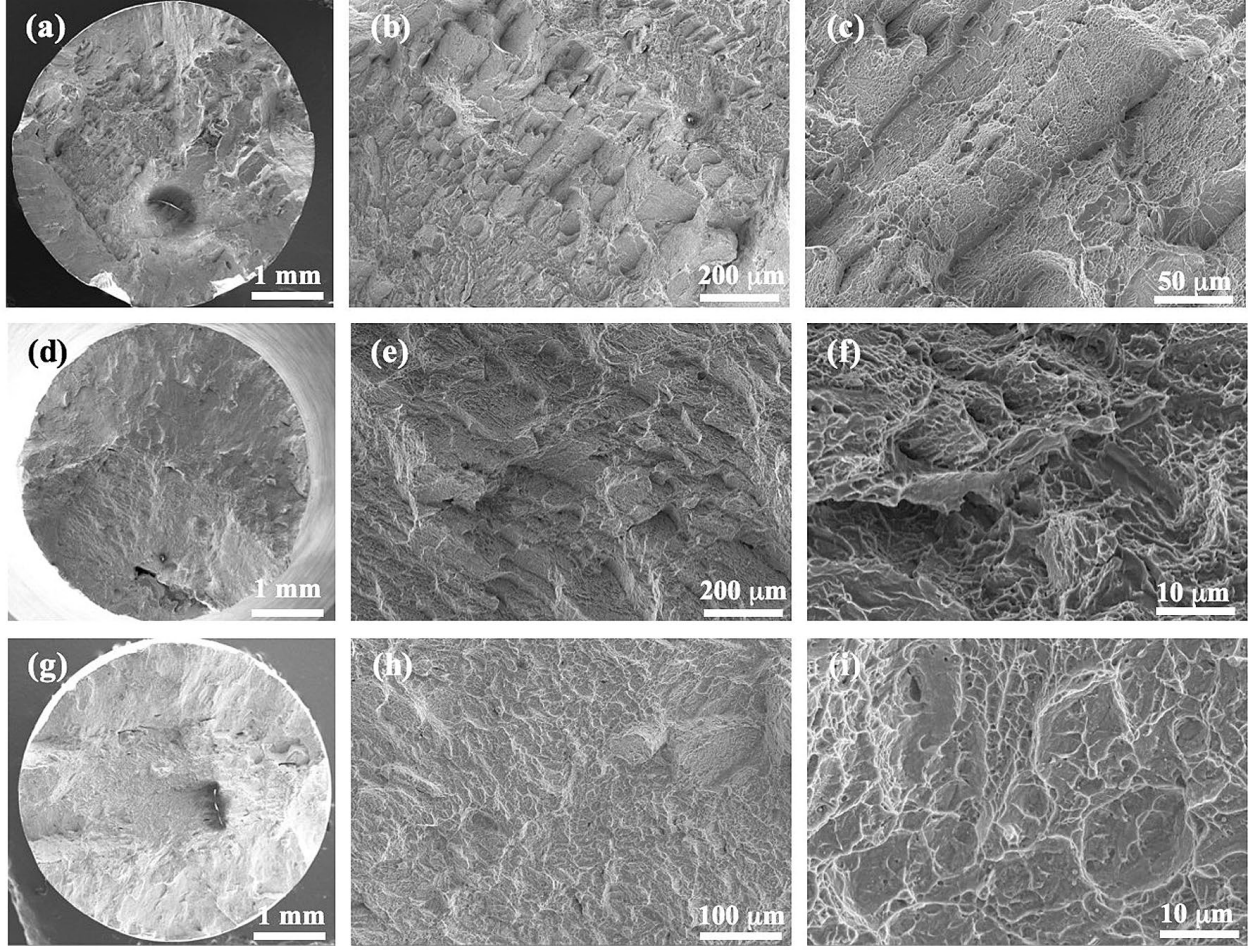
SEM micrographs showing the fracture surfaces of the samples made at 400 W (**a**–**c**) without reheating; with reheating at (**d**–**f**) 120 W and (**g**–**i**) 200 W.

Figure 10a shows the micro-hardness of matrix and band areas in the samples that were SLM-processed at 400 W and reheated at different laser powers. It is obvious that the matrix areas in all the samples show slightly higher micro-hardness than the (α + β)-containing band areas. The reheating laser power does not seem to show significant influence on micro-hardness. Figure 10b shows the tensile stress–strain curves for the samples that were processed at 400 W and reheated at different laser powers. It can be seen that the samples made without reheating show very high 0.2% yield strength (YS) and ultimate tensile strength (UTS) but very low elongation (EL < 4.5%). Samples that were made with laser reheating all show much better EL but relatively lower strengths, which is obviously due to the presence of (α + β)-bearing bands that are more ductile than α′. Among all the reheated samples, the one that was reheated at 120 W shows the best EL whereas the one reheated at 200 W which contains the thinnest (α + β)-bearing bands exhibits the lowest EL. In general, YS, UTS and EL do not change significantly with reheating laser power. Figure 11 shows the fracture surfaces of the tensile tested specimens. It is clear that the sample that was simply SLM-processed without laser reheating shows considerable large faceted and planar steps, a characteristic fracture mode of a martenstic needle structure in SLMed Ti-6Al-4V and also a typical brittle fracture mode. This type of steps is much smaller and rougher in the samples that have undergone layered laser reheating. Moreover, there are massive fine dimples prevailing over the fracture surfaces in these samples. All of these further confirm that the ductility of the reheated samples has indeed been considerably enhanced.

In comparison with tensile property data in previous studies (see Table 2), it is clear that the current SLMed Ti-6Al-4V samples that were laser reheated at 120 W show lower strengths but much higher EL as compared with simply SLMed samples^11,18^. The samples show remarkably higher strengths and comparable ductility relative to those SLMed + heat treated or SLMed + HIPed counterparts^18,21,22^. The samples also exhibit much higher strengths and comparable or better ductility as compared with Ti-6Al-4V samples that were made by EBM, DLD, or conventional methods^3,10,37,38^. The tensile properties of the samples are comparable to Xu et al.’s work^25,26^ which however involved complex and careful selection of a number of processing parameters as mentioned above. In summary, the current SLM + reheating/remelting strategy offers a simple but effective new route for engineering and tailoring of microstructure of SLM-processed metallic materials towards better mechanical properties.

**Table 2:** Comparison of tensile properties of current SLMed and reheated Ti-6Al-4V samples with those reported in previous studies.

Process	0.2% YS (MPa)	UTS (MPa)	El (%)	Microstructure	Ref
SLMed + reheated 120 W	1074	1169	9.0	Alternate αʹ and ultrafine α + β banding structures	Present study
SLMed	1202	1291	4.4	αʹ needle structure	Present study
SLMed	200	1300	6.0	αʹ needle structure	^11^
SLMed	1080	1250	5.0	αʹ needle structure	^18^
Slmed + heat treated	955	1004	12.8	Lamellar α + β structure	^21^
SLMed + heat treated	873	947	11.8	Lamellar α + β structure	^22^
SLM + HIPed	1000	1090	13	Lamellar α + β structure	^18^
EBMed	823	940	13.2	Basket-weave α + β structure	^10^
DLDed	960	1063	10.9	Lamellar α + β structure	^3^
Cast	847	976	5.1	Elongated α + intergranu1ar β	^37^
Wrought	945	979	10.0	Lamellar (α + β) among equiaxed α	^38^
SLMed	1112	1165	11.6	Ultrafine lamellar α + β structure	^25^

## Methods

Argon atomized Ti-6Al-4V powder with a particle size range of 15–53 μm was used in the current study. The powder was processed under a modulated pulsed laser mode using a Renishaw AM 400 system which is equipped with a ytterbium fiber laser and has a beam size of 70 μm in diameter^39^. Cubic samples with a dimension of 10 × 10 × 10 mm^3^ and elongated samples with a dimension of 60 × 10 × 12 mm^3^ were built in argon atmosphere for microstructural characterization and tensile testing, respectively. A Meander scanning strategy was used for hatch scanning on each layer^39^. A point distance (the distance between two adjacent point exposures in the scanning direction) of 60 μm and a hatch distance of 60 μm were used to process each powder layer. Each powder layer with a thickness of 60 μm was processed at a constant laser power of 400 W with an exposure time of 50 μs. After SLM processing of a powder layer, a laser reheating/re-melting was performed on the newly melted and solidified layer to tailor the microstructure in the new layer. Similarly, a Meander scanning strategy with a point distance of 60 μm and a hatch distance of 60 μm was adopted for the laser reheating. Different laser powers (30–200 W) with a constant exposure time of 50 μs were used for laser reheating for different samples to investigate the influence of laser reheating power on microstructure. After laser reheating, a new layer of powder would be delivered over the previous layer and then SLM processed and laser reheated. This alternated process continued on a layered basis until a complete sample was built.

The as-fabricated cubic samples were longitudinally sectioned by EDM (electrical discharge machining) and then ground using SiC papers from 200 grit up to 4000 grit before being polished using 3 μm diamond suspension and then colloidal silica suspension (or OPS solution)^39^. The samples were then etched in a solution containing distilled water, HNO_3_ and HF with a ratio of 10:5:1 prior to microstructural characterization using OM (optical microscopy) and SEM (scanning electron microscopy). A Leica DM4000 OM machine and a Zeiss Merlin SEM microscope were used for the microstructural characterization. TEM (transmission electron microscopy) study was also performed on some of the laser reheated samples. The TEM specimens were taken from the regions of interest using focused ion beam (FIB) from a FEI Helios G4 CX machine. The TEM experiments were carried out at an accelerating voltage of 200 kV using an FEI Tecnai F20 FEG TEM microscope^39^.

For tensile testing, the horizontally built samples were machined into cylindrical specimens with a parallel length of 23 mm and a nominal diameter of 4 mm along the parallel length^39^. Tensile tests were performed at room temperature following the ASTM E8 standard method^39^. The specimens were tested under strain rate control at 5 × 10^–4^/s until failure using a Zwick/Roell Z100 universal material testing machine^39^. After tensile testing, the fracture surfaces of the specimens were examined using SEM.

## Data Availability

The datasets generated during and/or analysed during the current study are available from the corresponding author on reasonable request.
